# Sex-biased severity of sarcoptic mange at the same biological cost in a sexually dimorphic ungulate

**DOI:** 10.1186/s13071-015-1186-6

**Published:** 2015-11-10

**Authors:** Jorge R. López-Olvera, Emmanuel Serrano, Anna Armenteros, Jesús M. Pérez, Paulino Fandos, João Carvalho, Roser Velarde, Francisco J. Cano-Manuel, Arián Ráez, José Espinosa, Ramón C. Soriguer, José E. Granados

**Affiliations:** Servei d’Ecopatologia de Fauna Salvatge (SEFaS), Departament de Medicina i Cirurgia Animals, Universitat Autònoma de Barcelona, Barcelona, Bellaterra E-08193 Spain; CESAM, Departamento de Biologia, Universidade de Aveiro, Campus Universitario de Santiago, Aveiro, 3810-193 Portugal; Departamento de Biología Animal, Biología Vegetal y Ecología, Universidad de Jaén, Campus Las Lagunillas, s.n., Jaén, E-23071 Spain; Agencia de Medio Ambiente y Agua, Isla de la Cartuja, Sevilla, E-41092 Spain; Espacio Natural Sierra Nevada, Carretera Antigua de Sierra Nevada, Km 7, Pinos Genil, Granada E-18071 Spain; Estación Biológica de Doñana (CSIC), Av. Américo Vespucio, s.n., Sevilla, E-41092 Spain

**Keywords:** Body condition, Kidney fat index, *Sarcoptes scabiei*, Sexual dimorphism, *Capra pyrenaica*

## Abstract

**Background:**

In sexually dimorphic species, male susceptibility to parasite infection and mortality is frequently higher than in females. The Iberian ibex (*Capra pyrenaica*) is a sexually dimorphic mountain ungulate endemic to the Iberian Peninsula commonly affected by sarcoptic mange, a chronic catabolic skin disease caused by *Sarcoptes scabiei*. Since 1992, sarcoptic mange affects the Iberian ibex population of the Sierra Nevada Natural Space (SNNS). This study aims at exploring whether mange severity, in terms of prevalence and its effects on body condition, is male-biased in Iberian ibex.

**Findings:**

One thousand and seventy-one adult Iberian ibexes (439 females and 632 males) were randomly shot-harvested in the SNNS from May 1995 to February 2008. Sarcoptic mange stage was classified as healthy, mildly infected or severely infected. Sex-biased prevalence of severe mange was evaluated by a Chi-square test whereas the interaction between mange severity and sex on body condition was assessed by additive models. Among scabietic individuals, the prevalence of severely affected males was 1.29 times higher than in females. On the other hand, both sexes were not able to take profit of a higher availability of seasonal food resources when sarcoptic, particularly in the severe stages.

**Conclusions:**

Sarcoptic mange severity is male-biased in Iberian ibex, though not mange effects on body condition. Behavioural, immunological and physiological characteristics of males may contribute to this partial sex-biased susceptibility to sarcoptic mange.

## Background

In sexually dimorphic vertebrates, males are more susceptible to become infected, experience more symptoms of infection than females and die earlier [1]. Such differences have been attributed to sex-specific differences in exposure or immune response [2]. However, infection intensity is often higher in males than in females even when external causes are controlled in experimental infections, which is mostly related to the well-established sex differences in immune functions. Androgens decrease male immune function, by reducing the activity of NK-cells and modulating the synthesis of pro- and anti-inflammatory cytokines, among other effects [3]. On the other hand, the larger body size of males supposes higher energetic requirements and makes males more prone to suffer from depletion of body reserves [4], and even to experience lower survival rates than females though only under poor environmental conditions [5], although an indirect role of parasites could also participate in such male-biased cost of life.

The Iberian ibex (*Capra pyrenaica*) is a medium-sized mountain ungulate with a marked sexual dimorphism in adult body size and weight [6]. Some 15,000 ibexes inhabit Sierra Nevada Natural Space (SNNS), a 172,315 hectares highly seasonal mountain area, with snowy winters (monthly mean temperature 0.5 °C and monthly precipitation 98.8 mm) usually followed by dry summers (monthly mean temperature 23 °C and monthly mean precipitation 35 mm). This seasonality affects ibex body condition, which is highest during summer and autumn and lowest during winter and spring, when food resources are limited. Such pattern is sex-dependent and fat reserves of male ibexes fall more than those of females in winter and spring [7]. Also, male and female Iberian ibexes live segregated outside the rutting period [8], which may suppose a different exposure to parasites for each sex [9].

Sarcoptic mange is a contagious skin disease caused by the mite *Sarcoptes scabiei*. In Iberian ibex, sarcoptic mange occurs as a chronic catabolic wasting disease which impairs body condition [10]. Sarcoptic mange usually reaches higher prevalence and severity during winter probably due to better environmental conditions for *Sarcoptes* [11], the increase in contact rates among individuals [12] and the poorer physical condition of the ibexes due to decreased food availability [7]. In 1992, a sarcoptic mange epizootic affected the ibex population of the SNNS [13]. Since then, sarcoptic mange prevalence has endemically persisted in the SNNS, showing a seasonal trend as described above.

The objective of this study is exploring whether sex differences in mange susceptibility and severity exist in Iberian ibex, a sexually dimorphic mountain ungulate. To achieve this goal, i) the proportion of mild and severe scabietic individuals for each sex, and ii) the impact of mange severity on the yearly fat reserves were analyzed.

## Methods

One thousand and seventy-one adult Iberian ibexes (439 females and 632 males), age ranging two to fifteen years, were shot-harvested by the SNNS staff from May 1995 to February 2008, as part of the SNNS ibex population management program (see [14] for the spatial distribution of the sampled ibexes within the SNNS). Sampling date was ranked from 1 (January 1st) to 365 (December 31st). The sex of the ibexes was determined visually and the age assessed by counting horn-segment counts [6]. The area of skin with sarcoptic mange lesions was visually estimated and the mange stage classified as healthy (no apparent lesions), mildly infected (skin surface affected up to 50 %) or severely infected (skin surface affected above 50 %), as previously described [15]. Later, ibexes were weighed to the nearest 0.1 kg. Both kidneys were collected with their associated fat, kept in plastic bags, transported to the laboratory in a cold box at 4 °C and finally weighed to the nearest 0.01 g.

The proportions of mildly and severely mange-infected ibexes for each sex were compared using a Chi-squared test. To explore whether sarcoptic mange effects on ibex body condition was sex-dependent, generalized additive models (GAM) were used [16]. Kidney fat reserves, a validated proxy to body condition [17], were considered the response variable, whereas Julian date (i.e., from 1 to 365), sex, mange severity as described above, and the two-way interactions were considered as the explanatory fixed factors. Since body condition follows a seasonal, age- and sex-dependent pattern in healthy Iberian ibex [7, 14], only the ibexes affected by mange (either mildly or severely) were used in the model, in order to detect the pure effects of sarcoptic mange on the seasonal cycle of body condition. Additive models were used because of the lack of linearity between date of sampling and body condition of ibexes in this population [7]. For descriptive statistics, however, we used kidney fat index (KFI). Though KFI has some statistical restrictions [17], it is related to kidney fat reserves [18] and hence useful for our descriptive purposes.

The best model was selected following the theoretic information approach based on Akaike’s Information Criterion (AIC). The models with the lowest AIC value were selected. Then, the remaining competing models according to their AIC value were ranked and their Akaike differences (∆i) with respect to the best model (lowest AIC) and the Akaike weight (W_i_) of each model were estimated [19]. Specific GAM requirements (e.g., homocedasticity, normality) were assessed before model interpretation [20]. All statistical analyses were performed using R software version 3.2.0 [21].

## Results and discussion

Among the mangy ibexes, the proportion of severely infected males was higher than females, which were rather mildly infected (X^2^ = 12.83, df = 1, p-value = 0.0003, Table 1). Such male-bias agrees with previously reported higher mange infection rate and higher parasitic load and diversity in males of other mountain ungulate species [22, 23]. Several factors could explain the higher proportion of severely infected males. Firstly, males could suffer from a higher exposition to ectoparasites, since adult males live in larger groups than females [24]. Male Iberian ibexes interact more not only with their counterparts, but also with objects in the area, and have a higher probability to acquire and maintain pseudoparasites, mostly during the rut [12, 25]. Secondly, male Iberian ibexes show a lower specific immune response to *Sarcoptes scabiei* [26], probably due to the immunosuppressive effects of testosterone [3] also reported in male Alpine ibex (*Capra ibex*) [27]. Although whether immune protection against sarcoptic mange depends on humoral or cellular response is controversial, sarcoptic infestation induces immunity (resistance) to reinfestation with elevated circulating antibody titres [28]. Moreover, healthy Iberian ibexes from areas where sarcoptic mange was present showed higher IgG values than scabietic ibexes [29], which seems to point to a certain relationship between the intensity of the immune humoral response and the resistance to develop clinical mange in this species. Finally, the lower body condition of males in certain seasons of the year [7] could also contribute to maintain a male-biased severity of mange into the wild. All these three factors would favor a faster evolution of sarcoptic mange to severe stages in males than in females after infection.

**Table 1:** Kidney Fat Index (KFI) values in healthy and *Sarcoptes scabiei*-infestated female and male ibexes from Sierra Nevada Natural Space (SNNS)

Mange category	Females			Males			TOTAL		
	n	Mean ± SD	Range	n	Mean ± SD	Range	n	Female	Male
Healthy	113 (25.7 %)	33.81 ± 2.52	0.56–72.91	278 (44.0 %)	24.35 ± 1.91	1.61–70.54	391 (36.5 %)	28.9 %	71.1 %
Mildly infested	194 (44.2 %)	17.01 ± 0.91	1.81–48.91	161 (25.5 %)	13.33 ± 0.69	1.71–58.67	355 (33.1 %)	54.6 %	45.4 %
Severely infested	132 (30.1 %)	9.12 ± 0.58	1.14–37.06	193 (30.5 %)	8.37 ± 0.63	1.35–44.64	325 (30.3 %)	40.6 %	59.4 %
TOTAL	439			632			1071		

**Fig. 1:**
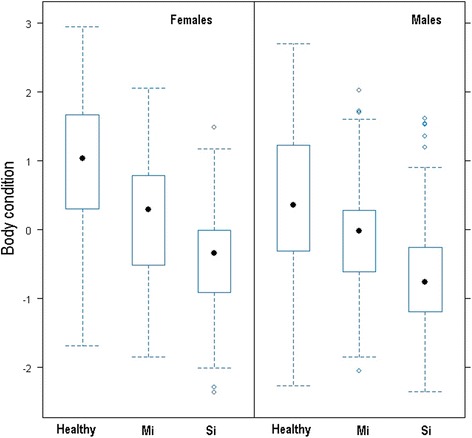
Body condition of the Iberian ibexes (*Capra pyrenaica*) sampled in the Sierra Nevada Natural Space according to their sex and sarcoptic mange status. Mi = Mildly infected; Si = Severely infected

**Fig. 2:**
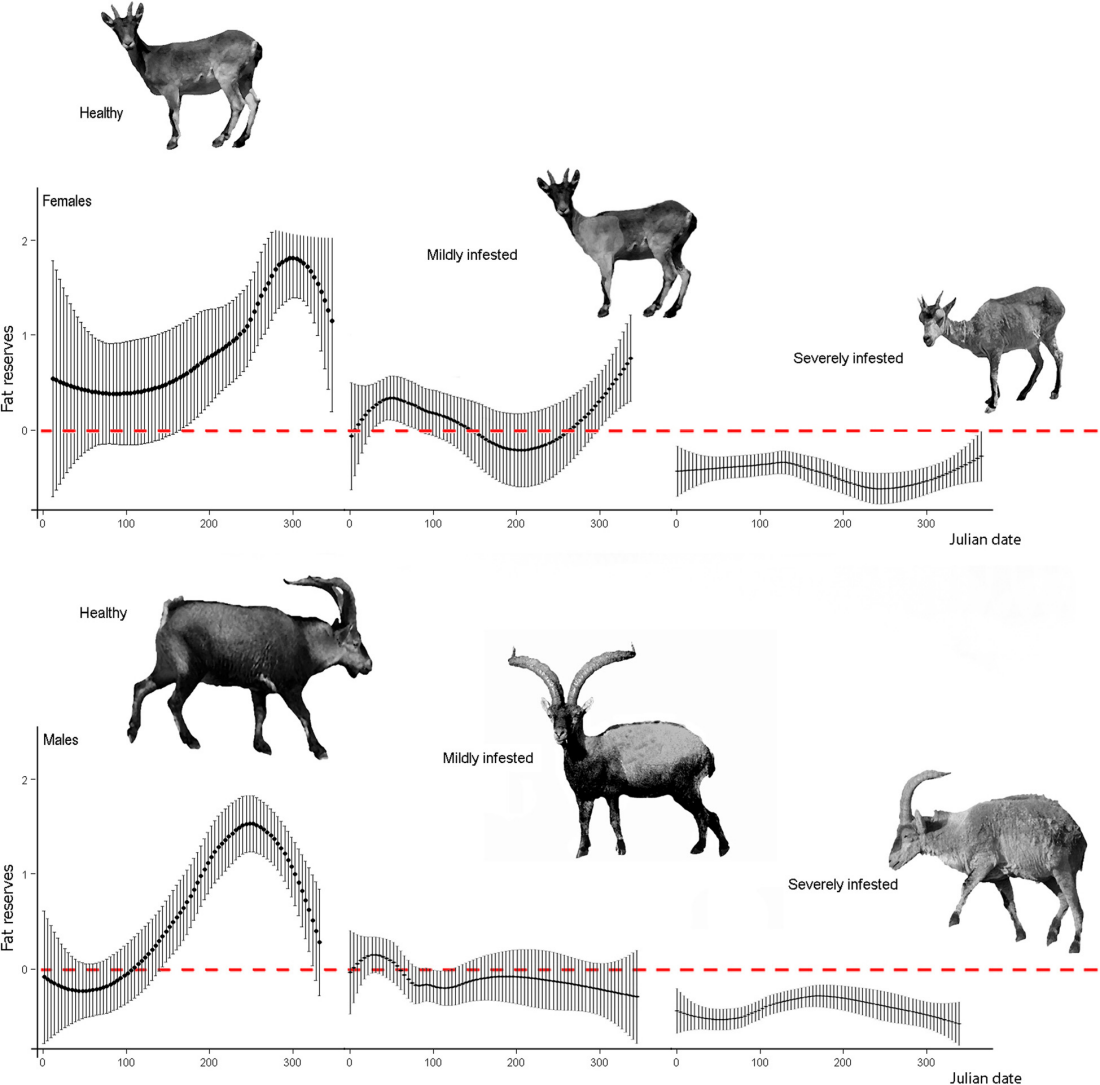
Seasonal evolution of kidney fat reserves in Iberian ibexes (*Capra pyrenaica*) sampled in the Sierra Nevada Natural Space according to their sex and sarcoptic mange status

Also, males showed a lower body condition than females independently of mange severity (β _males_ = −0.29, SE = 0.065, t-value = −4.62), but this difference between sexes decreased with increasing mange severity (Table 1 and Fig. 1). However, the impact of sarcoptic mange on kidney fat reserves (W _Mange severity + Sex_ = 0.73, R^2^ = 16 %, Tables 1 and 2) was not statistically different between sexes. In fact, the best-selected model included the additive effects of sex and mange severity (rather than the interaction of both factors) to explain body condition variability in Iberian ibexes from the SNNS (Table 2). According to this model, the severely infested ibexes had lower body condition (β _severely_ = −0.58, SE = 0.066, t-value = −8.82) than the mildly infested ones in both sexes. Body condition was also lower in both the mildly and severely infected ibexes as compared to the healthy ones (Table 1). Therefore, not only ibex body condition decreased with *S. scabiei* infestation, but also the intensity of the decrease was positively correlated with the increase in mange severity, as expected in such a chronic wasting disease. As sarcoptic mange severity increases, the capability of ibexes to energetically cope with mange decreases, and the severely affected ibexes showed a consistently low and constant body condition (Fig. 2), indicating that neither male and female ibexes are able to take profit of a higher availability of seasonal resources, particularly in the severe stages [14]. Body condition varies seasonally in healthy ibexes in the SNNS according to primary production, with higher body stores in the high primary production seasons (summer and autumn), and lower body condition when food resources are limited (winter and spring) [7]. Nevertheless, sarcoptic mange breaks such bottom-up regulation, mange effects on body condition overcoming the benefits of favorable environmental conditions and preventing the scabietic ibexes from taking advantage of high primary production in summer and autumn (Fig. 2; [14]).

**Table 2:** Model selection for assessing sexual differences in the effects of sarcoptic mange infection on body condition of Iberian ibexes

Biological model	K	AIC	Δi	*w*i
Mange severity + Sex	3	1259.1	0	0.73
Mange severity * Sex	4	1261.2	2.17	0.25
Date * Sex + Mange severity	11	1266.5	7.46	0.02
Date + Mange severity	5	1277.4	18.39	<0.01
Mange severity	3	1278.1	19.08	<0.01

K = number of parameters, AIC = Akaike Information Criterion, Δi = difference of AIC with respect to the best model, *w*i = Akaike weight. The best model is shown in bold. + indicates that the effects of the variables are additive, whereas * indicates the interaction of the variables

Although males were proportionally more severely affected, sarcoptic mange had a less pronounced effect on the body condition of male Iberian ibexes as compared to females. Conversely, and although male body condition was lower in all three mange categories than that of females, sex-related differences in body condition decreased with increasing mange severity (Table 1 and Fig. 1). Although mildly affected females seemed to retain a certain capability of increasing their body condition in fall (Fig. 2), such trend was not statistically significant according to the best model. Therefore, and as for seasonal trend [14], sarcoptic mange overwhelmed sex-effects on body condition (Table 1, Figs. 1 and 2).

To summarize, infected male Iberian ibex are more prone to develop severe mange than females, and the body condition of both males and females decreases progressively with increasing mange severity. Moreover, the negative effects of mange on body condition are so strong that any seasonal or sex-related pattern on body condition disappears, males and females following a similar trend in spite of males being more prone to reach the severe stages of the disease. Since neither sex is capable of profiting high primary production when severely infected, sexual differences in survival of mange-infected Iberian ibexes throughout the year should be related to factors other than seasonal variations in food availability, (e.g. ibex genetics or conditions of initial infestation among others).
